# The Treatment of Post-Partum Hæmorrhage

**Published:** 1894-03-31

**Authors:** 


### THE TREATMENT OF POST-PARTUM HÆMORRHAGE.

Among the various accidents and emergencies which a medical man has to treat, few make more demand upon his readiness and resource than does the occurrence of serious post-partum hæmorrhage. It may not often happen, nay, in well managed labours it is a very rare event, but its very rarity makes individual experience small, and so renders all the more interesting such a discussion as that which took place at the Harveian Society on February 15th, when Dr. Dakin undertook the task of summing up the means at the disposal of the accoucheur for treating cases of the sort. The main object of Dr. Dakin's paper was, however, to draw attention to two comparatively recent additions to our resources, viz., the intravenous injection of saline fluids and the plugging of the uterus. As regards the first, there seems no difference of opinion that, when hæmorrhage has been severe, and the heart tends to fail from want of something to contract upon, this proceeding is not only useful, but is the only thing by which the danger can be removed. In less severe conditions the problem is rather different, and it is probable that so long as there is blood enough in the vessels to ensure the continuance of the circulation, *i.e.*, to fill the auricles, set up contraction, lift the valves, and so enable the heart to go through its complete rhythm, there is little gain in injecting fluid, while, on the other hand, there is a chance of increasing the risk of hæmorrhage being set up afresh. When, however, the circulation begins to fail, and the fluttering pulse shows empty ventricles, even although the heart may seem to beat comparatively firmly, there should be no hesitation in immediately injecting a quantity of fluid into the veins. It must be remembered that women seldom die bleeding, and that the danger is not over because the hæmorrhage has ceased. It is the emptiness of the heart that causes death, and when there is blanching of the face, tossing of the arms, and want of breath, with failure or irregularity of pulse, the patient is in danger of death from hæmorrhage, even though there be not a trace of bleeding going on; and this danger can almost certainly be removed by the injection of saline fluid. The apparatus needed is very simple, and should be carried in every obstetric bag, viz., a knife, a pair of forceps, and a canula with four feet of indiarubber tubing attached.

Plugging the uterus is an operation the efficacy of which has been much discussed. To distend the uterus with a plug does not at first seem a very scientific proceeding, so long as we believe that its contraction and retraction are the principal means by which hæmorrhage is arrested. Nevertheless the results of the method are vouched for, and we are told that it is simple, safe, and successful. Iodoform gauze is said to be the best material, because, while bulky at first, it goes into a veiy much smaller space when the reviving uterus ultimately contracts upon it; but ordinary bandage or strips of sheeting, boiled in a saucepan if there is time, may be employed, the nurse rubbing in iodoform as they are passed in. One hand must be kept in the uterus, the cervix of which may, if necessary, be kept down by a vulsellum, and the strips of material must be passed in by means of a long pair of forceps, care being taken that the plugging begins at the upper part of the uterine cavity, and that each fresh piece is tied to the preceding one, so that they will unravel properly during extraction.

In the discussion Dr. Herman protested strongly against this treatment. He did not think any stress should be laid on certain German statistics of success, as the cases were probably slight ones which would have done equally well with much simpler treatment. He maintained that, unless the uterus contracted on the plug, no amount of stuffing which it was practically possible to introduce could press firmly enough to restrain hæmorrhage, while, if uterine contraction did occur, the plug would be unnecessary. He also said that the procedure involved certain dangers of its own, and, finally, that it was not always successful in its object. In those cases in which the uterus could not by other means be made to contract, he had no doubt at all that the proper treatment was to compress the uterus between the two hands and keep it compressed until such clotting and contraction should take place as to prevent recurrence of the bleeding. For this purpose the left hand should be introduced into the vagina, and the fingers bent into the palm so as to give a firm resisting surface, and the uterus should then be firmly pressed down in the anteflexed position by the right hand outside the abdomen, and firmly held between this and the fist within so long as may be necessary. If hæmorrhage came from a laceration of the cervix he thought it was best restrained by the application of a stitch.

The utility of plugging the uterus was, however, not without support among the speakers in the discussion, whatever might be its *modus operandi*, and it was pointed out that immediate stitching of a lacerated cervix was by no means a simple operation amid the surroundings in which confinements often took place, and that, at any rate in hæmorrhage from this source, plugging with iodoform gauze or wool was an effectual hæmostatic.

On the whole, it would seem as if Dr. Herman were in the right, and that, at the least, continuous compression should be resorted to before plugging. Nevertheless, if we are to believe, as we almost must do, that the uterus may contract irregularly and partially, and that, for example, atony may persist over the placental site after fair contraction has been set up in other parts, it does not seem unreasonable to think that the presence of a plug might make even such a partial contraction effectual, as without it might be quite unable to bring pressure to bear upon the soft spot.

The principal means at our disposal then in the prevention and treatment of post-partum hæmorrhage are (*a*) proper conduct of the third stage of labour, (*b*) direct stimulation of the uterus by kneading by the hand outside; (*c*) the same by the hand inside, combined with search for portions of placenta, or membranes, or clots; (*d*) ergot, for the action of which, however, there is no time to wait; (*e*) continuous compression; (*f*) injection of hot water into the uterus; (*g*) plugging of the uterus; while all along, if the integrity of the circulation is endangered, intravenous injection of saline fluid may be used to promote recovery or to prevent the patient from dying in our hands.

